# Diagnosis, pathophysiology and preventive strategies for cardiac surgery-associated acute kidney injury: a narrative review

**DOI:** 10.1186/s40001-023-00990-2

**Published:** 2023-01-24

**Authors:** Ying Yu, Chenning Li, Shuainan Zhu, Lin Jin, Yan Hu, Xiaomin Ling, Changhong Miao, Kefang Guo

**Affiliations:** Department of Anaesthesiology, Zhongshan Hospital, Fudan University, No 180 Fenglin Road, Xuhui District, Shanghai, 20032 China

**Keywords:** Acute kidney injury, Cardiac surgery, Diagnosis, Pathophysiology, Pathogenesis, Prevention

## Abstract

Acute kidney injury (AKI) is a common and serious complication of cardiac surgery and is associated with increased mortality and morbidity, accompanied by a substantial economic burden. The pathogenesis of cardiac surgery-associated acute kidney injury (CSA-AKI) is multifactorial and complex, with a variety of pathophysiological theories. In addition to the existing diagnostic criteria, the exploration and validation of biomarkers is the focus of research in the field of CSA-AKI diagnosis. Prevention remains the key to the management of CSA-AKI, and common strategies include maintenance of renal perfusion, individualized blood pressure targets, balanced fluid management, goal-directed oxygen delivery, and avoidance of nephrotoxins. This article reviews the pathogenesis, definition and diagnosis, and pharmacological and nonpharmacological prevention strategies of AKI in cardiac surgical patients.

## Introduction

More than 2 million cardiac surgeries are performed worldwide each year, and acute kidney injury (AKI) is a serious complication after cardiac surgery, the second leading cause of AKI in the intensive care unit (ICU). The incidence of acute kidney injury after cardiac surgery (CSA-AKI) is as high as 40%, and approximately 3% of patients require at least temporary renal replacement therapy (RRT) [1]. Patients with severe AKI are confronted with a 3- to eightfold higher perioperative mortality, a prolonged length of ICU and hospital stay, and an increased cost of health care [2]. CSA-AKI is associated with increased long-term mortality, with 5-year and 7-year cohort survival rates of 54% and 38%, respectively [3]. Approximately 25% of patients with AKI will suffer from chronic kidney disease (CKD) after a three-year period [4]. The economic impacts of CSA-AKI are phenomenal. The hospitalization costs were as high as USD $26,000 in patients with AKI not requiring RRT and exceeded USD $69,000 for AKI requiring RRT, leading to a total annual cost for CSA-AKI of almost USD $1 billion in the United States [5].

This narrative review assesses the pathophysiology of AKI, classification and definition, diagnostic criteria and available novel biomarkers, as well as the commonly used and promising prevention strategies in cardiac surgical patients.

## Definition and diagnosis

### Definition

Cardiorenal syndrome (CRS) includes a range of diseases involving the heart and kidneys, in which acute or chronic dysfunction in one organ can lead to acute or chronic dysfunction in the other. Ronco presented a new classification of CRS with 5 subtypes that reflect the pathophysiology, the timeframe, and the nature of concomitant cardiac and renal dysfunction [6]. Subsequently, the American Heart Association published a detailed scientific statement covering its pathophysiology, diagnosis, and management [7]. According to the CRS classification definition, CSA-AKI can be classified as type 1 CRS (acute CRS), which is characterized by a rapid decline of cardiac function, leading to AKI [6]. Despite this cardiorenal theoretical framework, there is no consensus on the definition of CSA-AKI, and the definition and diagnosis of CSA-AKI in the literature are derived from AKI.

The current definition of AKI, according to (Kidney Disease: Improving Global Outcomes) KDIGO [8], stipulates an increase in serum creatinine (SCr) by at least 26.4 μmol/L within 48 h, an increase in SCr to 1.5 times baseline that is known or presumed to have occurred within the previous 7 days, or a urine volume of less than 0.5 mL/kg per hour for 6 h. A sustained increase in creatinine for more than 3 months is labelled CKD. The concept of acute kidney disease (AKD) is intended to bridge the gap between AKI and CKD, which refers to a sustained alteration in kidney function over a 3 month period [9].

### Diagnosis of CSA-AKI

In 2004, the Acute Dialysis Quality Initiative (ADQI) proposed a classification for acute renal failure (ARF; this term was later replaced by AKI) based on the patient SCr, urine output, glomerular filtration rate (GFR), and clinical outcome: the Risk, Injury, Failure, Loss of kidney function, and End-stage kidney disease (RIFLE) classification [10]. In 2007, the Acute Kidney Injury Network (AKIN) proposed a revised version of AKI classification [11] to improve the sensitivity of AKI criteria, which made several changes: an absolute increase in SCr was added as a criterion, patients starting RRT were classified as stage 3, and changes in GFR and clinical outcome stages were removed. AKIN proposed that although the diagnosis of AKI is based on the SCr changes over the course of 48 h, staging occurs over a 1-week time frame. Based on the two previous classifications, the 2012 Global Working Group on Improving Outcomes in Acute Kidney Injury proposed the KDIGO classification [8] with the aim of harmonizing the definition of AKI. KDIGO adopted the stages proposed by AKIN but removed the acute rise of SCr over 0.5 mg/dL in stage 3 (Table 1).

**Table 1:** The classification and staging of the RIFLE, AKIN and KDIGO criteria

Workgroup	RIFLE	AKIN	KDIGO
	Acute dialysis quality initiative group	Acute kidney injury Network	Kidney disease improving Global outcome
Criteria	SCr, UO, GFR, clinical outcome	SCr, UO	SCr, UO, GFR
Staging	**Risk** SCr increased by 1.5 × UO < 0.5 mL/kg/h for 6 h eGFR decreased by > 25% within 7 d	**Stage 1** SCr increased > 0.3 mg/dL SCr increased by 1.5 × to 2.0 × UO < 0.5 mL/kg/h for > 6 h	**Stage 1** SCr increased ≥ 0.3 mg/dL within 48 h SCr increased by 1.5 × to 1.9 × , which is known or presumed to have occurred within prior 7 d UO < 0.5 mL/kg/h for 6–12 h
	**Injury** SCr increased by 2.0 × UO < 0.5 mL/kg/h for 12 h eGFR decreased by > 50%	**Stage 2** SCr increased by > 2.0 × to 3.0 × UO < 0.5 mL/kg/h for > 12 h	**Stage 2** SCr increased by 2.0 × to 2.9 × UO < 0.5 mL/kg/h for 12 h
	**Failure** SCr increased by 3.0 × SCr ≥ 4 mg/dL with acute rise ≥ 0.5 mg/dL UO < 0.3 mL/kg/h for 24 h Anuria for 12 h eGFR decreased by > 75%	**Stage 3** SCr increased by > 3.0 × SCr ≥ 4 mg/dL with acute rise ≥ 0.5 mg/dL UO < 0.3 mL/kg/h for 24 h Anuria for 12 h	**Stage 3** SCr increased by ≥ 3.0 × SCr ≥ 4 mg/dL Initiation of RRT UO < 0.3 mL/kg/h for 24 h Anuria for 12 h eGFR < 35 mL/min/1.73 m^2^(in patients < 18 yr)
	**Loss** Complete loss of kidney function > 4 wk		
	**End-stage kidney disease** Complete loss of kidney function > 3 mo		

*AKI* acute kidney injury, *GFR* glomerular filtration rate, *SCr* serum creatinine, *RRT* renal replacement therapy, *UO* urine output, *×(symbol)* times baseline

In the published literature on CSA-AKI, all three diagnostic criteria have been adopted [12]. Luo et al. prospectively analysed a clinical database of 3,107 adult patients who were admitted to the ICU and found that compared with the RIFLE criteria, KDIGO was more predictive of in-hospital mortality, but there was no significant difference between AKIN and KDIGO [13]. Yaqub et al. retrospectively reviewed data from 1508 patients who underwent isolated (Coronary artery bypass grafting) CABG surgery and found that the performances of the AKIN and KDIGO criteria were comparable in diagnosing AKI, whilst the RIFLE definition, although overestimating the incidence of AKI, had a better power to predict mortality than the other two definitions [14].

### Biomarkers of CSA-AKI

Early recognition and diagnosis of AKI are critical because prevention before clinical manifestation may provide better outcomes than treating patients with established AKI. However, the current diagnostic criteria are not suitable for the timely diagnosis of AKI because neither the decrease in urine output nor the increase in SCr is recognizable early. SCr level is a traditional biomarker of renal injury, but it can be affected not only by physiological processes (for example, urinary creatinine clearance or muscle mass) but also by drugs and potential comorbidities. In addition, SCr concentration is influenced by sex, age, and protein intake. Meanwhile, the calculation of postoperative urine output is largely limited by intraoperative fluid therapy and diuretic drugs.

When the kidneys are attacked, molecular changes follow, leading to cellular damage. This results in cells expressing and releasing multiple cellular markers called biomarkers. Research efforts have been focussed on finding an indicator of early renal injury to quickly diagnose it with high specificity and sensitivity. Evidence has demonstrated that biomarkers are present before the onset of AKI as currently diagnosed and therefore have potential advantages in the early diagnosis of AKI. More than 60 urine and blood biomarkers have been tested as indicators of renal injury, and elevation of these biomarkers precedes increases in SCr concentrations [15].

In CSA-AKI, several biomarkers, have been identified and validated [16]. Neutrophil gelatinase-associated lipoprotein (NGAL) is an acute-phase reactive protein expressed by proximal tubule epithelium and neutrophils. NGAL is released into the bloodstream after tubular cell injury occurs, and an increase from baseline can be detected within two hours after injury occurs. A recent meta-analysis reported the AUCs for urinary and for plasma NGAL of 0.75 (95% CI 0.73–0.76) and 0.80 (95% CI 0.79–0.81) for severe AKI. Albert et al. suggested the cutoff concentrations at 95% specificity for urinary NGAL were > 580 ng/mL (sensitivity: 27%), and for plasma NGAL were > 364 ng/mL (sensitivity: 44%) [17].

Kidney injury molecule 1 (KIM-1) is a transmembrane glycoprotein that is up-regulated in proximal renal tubular cells after ischaemia–reperfusion injury. A meta-analysis reported a sensitivity of 0.73 (95% CI 0.45–0.93) and a specificity of 0.77(95% CI 0.62–0.90) for predicting AKI in the cardiac surgery population, but no consistent diagnostic threshold has been established [18].

Dickkopf-3 (DKK3) is a glycoprotein that is a stress-induced pro-fibrotic molecule derived from renal epithelial cells. It can promote renal tubulointerstitial fibrosis by regulating the Wnt/β-catenin signalling pathway [19]. The AUC for predicting postoperative AKI was 0.783 (95%CI 0.747–0.820). Schunk et al. found that urine DKK3/ creatinine ratio > 471 pg/mg on admission was associated with a significantly increased risk of CSA-AKI, which could be used for preoperative screening of patients at high risk for CSA-AKI [20].

Tissue inhibitor of metalloproteinases-2 (TIMP-2) and Insulin-like growth factor-binding protein 7 (IGFBP7) are two molecules involved in G1 cell cycle arrest. In early response to ischaemia–reperfusion injury, to allow repair and avoid inflammation and cell death, renal tubule cells enter G1 cell cycle arrest, where IGFBP7 and TIMP-2 products increased [21]. The peak concentration of [TIMP-2] × [IGFBP7] appeared at 4 h after CPB. Meta-analysis showed a pooled sensitivity of 0.79 (95%CI 0.71–0.86), a specificity of 0.76 (95%CI 0.72–0.80), and an AUC of 0.868. Although the cutoff values of urinary TIMP-2 and IGFBP7 for the early prediction of CSA-AKI varied across the studies, > 0.3 (ng/mL)^2^/1000 was the most commonly used [22].

Several novel biomarkers also showed moderate discrimination in patients that underwent cardiac surgery. Liver fatty acid binding protein (L-FABP) is normally produced by tissues such as the liver and is released into the blood. In renal tubular injury, L-FABP is secreted into the urine due to incomplete lipid binding, and urinary L-FABP is considered as a potential biomarker for AKI. Corrected with CPB duration as a covariate factor, urinary L-FABP at 16–18 h after cardiac surgery can well predict AKI within 7 days after surgery, with an AUC of 0.742 [23]. Neprilysin is a single-pass membrane glycoprotein and it is widely distributed in renal brush border. During kidney injury, neprilysin is shed from the tubular brush edge of the kidney and is excreted, which can be detected in urine. The discriminatory power of neprilysin for detecting POD 1 AKI corresponded to an AUC of 0.77 (95% CI 0.65–0.90) [24].

The PrevAKI multi-centre trial confirmed that after identifying high-risk AKI patients defined as [TIMP-2] × [IGFBP7] ≥ 0.3 (Nephrocheck®), the application of care bundle can reduce the incidence of stage 2 and 3 AKI after cardiac surgery [25]. NephroCheck^®^ is a commercially available point-of-care test to measure [TIMP-2] × [IGFBP7]. Single-centre studies have proposed that the introduction of [TIMP-2] × [IGFBP7] as a new tool for identifying AKI risk may result in cost savings for hospitals [26]. However, the current evidence is insufficient to compare the cost-effectiveness of NephroCheck^®^ and other biomarker detection tools due to limited data and lack of uniform cutoff values [27].

At present, the diagnostic efficacy of most biomarkers in predicting CSA-AKI needs to be improved, and there is no uniform diagnostic cutoff value for most biomarkers. Moreover, whether early identification of high-risk patients based on the biomarkers can improve patient outcomes or alleviate medical burden needs to be confirmed by more studies. Nevertheless, biomarkers have received widespread attention, and the ADQI proposed the inclusion of biomarker in KDIGO staging [28].

### Point-of-care ultrasound

Renal ultrasound to evaluate renal function after cardiac surgery is also a hot field of recent research. It is believed that the measurement of perioperative renal artery or venous index can reflect the status of renal blood circulation, which is promising to help early identification and prediction of CSA-AKI [29].

#### Arterial profile: renal artery

Renal arterial Resistance Index (RRI) is a method of measuring pulse blood flow, which reflects the blood flow resistance caused by the distal microvascular bed at the measurement site. RRI can be calculated by measuring the flow velocity of intralobar artery or arcuate artery. The specific formula is as follows: RRI = (systolic velocity—diastolic velocity)/systolic velocity. A prospective observational study using TEE to measure RRI to assess the occurrence of postoperative AKI after CABG suggested that increased intraoperative RRI was an independent predictor of postoperative AKI, with a cutoff of > 0.68 and an AUC of 0.705 (95%CI 0.588–0.826) [30].

Intraparenchymal renal resistive index variation (IRRIV) is an index derived from RRI, which is the percentage of RRI decline from baseline level after abdominal compression (10% of the subject's body weight). The pilot study by Samoni et al. found IRRIV showed a good correlation with renal function reserve, with a sensitivity and specificity of 100% and 84%, respectively, and AUC of 0.80 (95%CI 0.64–0.96) as diagnostic performance. The sensitivity and specificity of IRRIV in predicting subclinical AKI after cardiac surgery were 46.1% and 100%, respectively, and the AUC was 0.73 (95%CI 0.58–0.87) [31].

#### Venous profile: renal, hepatic, and portal veins

High CVP secondary to venous congestion has been shown to be associated with increased postoperative mortality. However, the change of CVP is not only dependent on the degree of venous filling, but also affected by intrathoracic pressure or right ventricular diastolic function. Ultrasound analysis based on the waveform of organ veins can help to identify the presence of renal venous congestion [32].

Since the pressure of the right atrium is transmitted to the abdominal veins throughout the cardiac cycle, the venous waveform obtained by pulsed-wave Doppler will have systolic and diastolic components, and the amplitude of the waveform is the blood flow velocity. Under physiological conditions, the blood flow waveform in the renal vein is continuous throughout the cardiac cycle, that is, the waveform is uninterrupted. In the case of mild venous congestion, the systolic component and diastolic component appeared, forming biphasic waveform. In severe venous congestion, only the diastolic component appeared, forming monophasic waveform. Patterns of intrarenal vein flow have been found to be strongly associated with adverse clinical outcomes and independently predict increased risk of perioperative AKI and mortality [33].

In the normal hepatic venous blood flow waveform, the amplitude of systolic component is higher than that of diastolic component. For mild venous congestion, the amplitude of systolic component is lower than diastolic component. In severe congestion, there is a retrograde systolic component (opposite to the diastolic component). Abnormal postoperative hepatic vein blood flow waveform is associated with the occurrence of CSA-AKI [34]. In addition, a recent study suggests that the preoperative ratio of VTI of the retrograde and anterograde waves of the hepatic veins is independently correlated with increased creatinine levels after cardiac surgery [35].

Portal blood flow is often less susceptible due to the buffering effect of the hepatic sinusoids, that is, the flow waveform is continuous. Abnormal blood flow is indicated when the diastolic and systolic flow rates are different.

Due to the buffering effect of hepatic sinuses, the portal vein flow is often not susceptible, that is, the flow waveform is continuous. Abnormal blood flow is indicated when the diastolic and systolic flow rates are different. Specifically, a portal pulse rate, calculated as follows: (systolic velocity—diastolic velocity)/systolic velocity, > 50% is considered a sign of venous congestion. It was found to be an independent predictor of CSA-AKI along with the pattern of intrarenal vein flow [36].

### Risk factors and stratification tools

A range of perioperative risk factors for CSA-AKI have been identified (Table 2), including patient-related, anaesthesia-related, surgical procedure-related, and CPB-related factors [37–39]. Several risk scores based on these factors have been developed (Table 3). After the introduction of traditional clinical scores [40–43], new scores have applied various methods to improve predictive performance, such as adding biomarkers [44, 45], laboratory test-based [46, 47], and using machine learning technology [48] to build predictive models. These scores have been reported with diagnostic accuracy in the range of 70–85%. Furthermore, most of these data were come from retrospective studies. Therefore, larger scale, multicentre, and prospective studies are needed to further explore their effectiveness in different clinical scenarios and situations.

**Table 2:** Common risk factors for AKI after cardias surgery, adapted from [37–39]

	Patient-related	Procedure-related (surgery, anaesthesia, CPB-related)
Preoperative	•Gender •Advanced age •Severe cardiac disease •Previous cardiac surgery •Active congestive heart failure •Cardiogenic shock •NYHA class III/IV •Left ventricular ejection fraction < 35% •Left main coronary artery disease •Anaemia •Coexisting disease (Peripheral vascular disease, hypertension, generalized atherosclerotic disease, chronic obstructive pulmonary disease, previous cerebrovascular accidents, diabetes mellitus, chronic kidney disease, chronic liver disease) •Nephrotoxins (ACEis/ARBs, antibiotics, diuretics, or NSAIDs)	•Preoperative contrast media exposure •Preoperative insertion of intra-aortic balloon pump •Emergency status
Intraoperative		•Type of surgery (valvular, valvular and coronary, emergency and redo surgery) •CPB (non-pulsatile, low-flow, low-pressure perfusion) •Hypotension •Hypothermia •Deep hypothermic circulatory arrest CPB duration •Cross-clamp duration •Anaemia (Haemodilution, Haemolysis) •Transfusion load •Embolism
Postoperative		•Low cardiac output •Hypovolemia •Hypotension •Intense vasoconstriction •Atheroembolism (requiring Intra-aortic balloon pump) •Sepsis •Nephrotoxins •Cardiogenic Shock

*ACEis* angiotensin-converting enzyme inhibitors;, *AKI* acute kidney injury, *ARBs* angiotensin receptor blockers, *CPB* cardiopulmonary bypass, *NSAIDs* nonsteroidal anti-inflammatory drugs, *NYHA* New York heart association

**Table 3:** Several models for AKI after cardiac surgery

Model	Risk factors included	AUC/C-statistic in first-time report	Reference
Thakar et al./Cleveland Clinic score (2005)	•Female gender •Congestive heart failure •Left ventricular ejection fraction < 35% •Preoperative use of intra-aortic balloon pump •Chronic obstructive pulmonary disease •Insulin-requiring diabetes •Previous cardiac surgery •Emergency surgery •Type of surgery •Preoperative creatinine	0.81 for ARF requiring dialysis	[40]
Mehta et al. (2006)	•Glomerular filtration rate •Preoperative serum creatinine •Type of surgery •Age •Diabetes •Chronic lung disease •Myocardial infarction •Cardiogenic shock •NYHA status •Race •Previous cardiovascular surgery •Female gender •Peripheral or cerebrovascular disease •Body surface area •Left ventricular ejection fraction •Emergent status •Triple-vessel disease •Left main disease •Prior percutaneous coronary interventions •Hypertension •Immunosuppressive treatment •Aortic stenosis •Mitral insufficiency	0.84 (0.83 in simplified model) for AKI requiring RRT	[41]
Birnie et al./Leicester Score (2014)	•Age •Renal function (Cockroft–Gault formula) •Diabetes mellitus •NYHA status •Left ventricular function •Elective/urgent/emergency surgery •Type of surgery •BMI •Smoking habit •Hypertension •Peripheral vascular disease •Preoperative haemoglobin •Triple-vessel disease •Time from catheterism to surgery	0.74 for any stage AKI 0.79 for KIDGO stage 3 AKI	[42]
Coulson et al. (2020)	Preoperative model for AKI: •Preoperative haemoglobin •Preoperative creatinine •Age •NYHA status •BMI Postoperative model for AKI •Preoperative haemoglobin •Preoperative creatinine •Perfusion time •NYHA status •BMI Preoperative model for RRT: •Preoperative creatinine •Previous cardiac surgery •NYHA status •Type of surgery Postoperative model for RRT: •Perfusion time •Preoperative creatinine •Intra-aortic balloon pump	0.68 for preoperative model for AKI 0.70 for postoperative model for AKI 0.80 for preoperative model for RRT 0.85 for postoperative model for RRT	[43]
Wang et al. (2022)	•Postoperative creatinine •Aortic cross-clamping time •Emergency surgery •Preoperative cystatin C	c-statistic of 0.851 for AKI requiring RRT	[44]
Demirjian et al. (2022)	•Preoperative serum creatinine •Postoperative serum creatinine •Postoperative serum albumin •Postoperative blood urea nitrogen •Postoperative serum potassium •Postoperative serum sodium •Postoperative serum bicarbonate	0.876 for moderate to severe AKI (KIDGO stage 2 or 3) within 72 h after cardiac surgery 0.854 for moderate to severe AKI within 14 days 0.916 for AKI requiring dialysis within 72 h 0.900 for AKI requiring dialysis within 14 days	[46]
Chen et al. (2020)	•Interferon-γ •Interleukin-16 •Mip-1α (macrophage inflammatory protein-1α)	C-statistic of 0.87 for severe AKI (AKIN stage 2 or 3)	[47]
Zhang et al.(2022)	•Age •Male •Preoperative serum creatinine •Preoperative neutrophil to lymphocyte ratio •Preoperative blood glucose •Preoperative high-density lipoprotein •Intraoperative urine output •Conventional ultrafiltration •Central venous pressure •Perfusion flow •Intubated PaO_2_/FiO_2_ ratio •Postoperative haemoglobin •Postoperative serum potassium •Postoperative lactic dehydrogenase	0.824	[48]

*AKI* acute kidney injury, *ARF* acute renal failure, *BMI* body mass index, *FiO*_*2*_ inspired oxygen fraction, *NYHA* New York heart association, *PaO*_*2*_ partial pressure of oxygen, *RRT* renal replacement therapy

## Theories of CSA-AKI pathophysiology

The pathophysiology of CSA-AKI is multifactorial and thus far is not fully understood. Several major pathways may be involved in the development of CSA-AKI, including renal hypoperfusion, ischaemia‒reperfusion injury (IRI), activation of the inflammatory cascade, oxidative stress, nephrotoxin exposure, and genetic polymorphisms, which can occur at any time during the perioperative period.

### Renal hypoperfusion and reperfusion injury

The renal medulla has high metabolic demands but lower partial oxygen pressure (PaO_2_) than the rest of the kidney (10 to 20 mmHg) [49], making it vulnerable to hypoxic injury. Therefore, renal hypoperfusion is usually the initial factor of CSA-AKI. In cardiac surgery, cardiopulmonary bypass (CPB)-related low flow, low pressure, non-pulsatile perfusion [50], haemodilution, emboli [51], rewarming [52] and intravascular haemolysis [53] are all risk factors for renal ischaemia. Continuous renal ischaemia may lead to damage to tubular structures and tubular epithelial cells, resulting in tubular dysfunction. Renal hypoperfusion and tubular cell injury are accompanied by oxidative damage and inflammatory events [54].

The low cardiac output is a common cause of early postoperative AKI [55]. Prolonged low cardiac output or hypotension leads to the activation of the sympathetic nervous system and the renin–angiotensin–aldosterone system, resulting in systemic vasoconstriction, which reduces renal blood flow and leads to renal damage [49]. Apart from arterial perfusion pressure, obstruction of return attributing to venous congestion is another haemodynamic determinant of renal insufficiency during cardiac surgery [56]. Evidence continues to support that perioperative high venous pressure is an independent risk factor for CSA-AKI [57], even more important than hypotension [58]. To ensure adequate renal blood flow and glomerular filtration, the difference between arterial driving pressure and venous outflow pressure must be maintained sufficiently large. Increased central venous pressure due to various causes, such as excessive fluid therapy or tricuspid valve disease, will lead to renal venous hypertension, increasing renal resistance, and ultimately impinging renal perfusion. At the beginning of the decrease in renal arterial perfusion, the renin level increases, causing the contraction of the efferent arterioles and the increase of glomerular pressure; thus, maintaining the filtration rate. Later, when renal venous pressure increases and renal blood flow decreases, the compensatory increase in filtration rate is destroyed, GFR decreases, and renal injury is aggravated [7].

The essence of renal hypoperfusion injury is insufficient tissue oxygen supply. Oxygen delivery to the renal medulla is a complex interactive process involving pressure, flow, and oxygen content. According to an increasing number of studies, the kidneys may suffer from an imbalance in oxygen supply and demand during CPB. Ranucci et al. showed that the nadir of oxygen supply during CPB, with a critical threshold of < 272 mL/min/m^2^, was the best predictor of AKI after cardiac surgery [59]. The effects of decreased haemoglobin concentration and haemodilution during CPB on the incidence of AKI are probably due to decreased oxygen-carrying capacity. At the start of CPB, haemodilution occurs as an inevitable result of mixing the patient's blood with the prefilling fluid in the CPB circuit, but it is believed that reduced blood viscosity and improved microcirculation perfusion may attenuate the risk of reduced blood oxygen-carrying capacity, and oxygen supply is prone to meet the demands of tissue metabolism [60]. Although moderate haemodilution to a haematocrit of less than 25% is acceptable, extreme haemodilution to a haematocrit of less than 21% or transfusion are not desirable, both of which are associated with AKI and increased risk of dialysis [61]. The elements of oxygen delivery during CPB include pump flow, haemoglobin, oxygen saturation, and arterial oxygen tension [62]. The use of goal-directed oxygen delivery during CPB has been proven to reduce CSA-AKI by approximately 50% [63].

In addition to CPB-induced changes, a low cardiac output is also a common cause of early postoperative AKI [55]. Prolonged low cardiac output or hypotension leads to activation of the sympathetic nervous system (SNS) and the renin–angiotensin–aldosterone system (RAAS), resulting in systemic vasoconstriction, which reduces renal blood flow and leads to renal damage [49]. Apart from arterial perfusion pressure, obstruction of return attributed to venous congestion is another haemodynamic determinant of renal insufficiency during cardiac surgery [56]. In addition, bleeding complications and inflammatory responses, which are common during cardiac surgery, may contribute to renal hypoperfusion.

Renal perfusion may improve after CPB; however, IRI may subsequently occur and may lead to the opening of mitochondrial permeability transition pores in the kidney [64], regulating mitophagy [65] and thereby leading to AKI. IRI can also increase the production of reactive oxygen species (ROS) [65], which can induce inflammation and promote the occurrence of AKI.

### Inflammation and oxidative stress

During cardiac surgery, systemic inflammation occurs as a result of the contact of blood components with the surface of the CPB circuit, IRI, and oxidative damage. The inflammatory response consists of activation of the vascular endothelium and immune system and release and recruitment of proinflammatory cytokines and free radicals, contributing to tubular damage [66]. Due to biological incompatibility, CPB circuit materials provide a significant stimulus for proinflammatory pathways, activation of the complement system, and haemolysis [67]. An increase in the level of proinflammatory cytokines is observed in CPB surgery when compared with that in off-pump surgery [68]. Increased postoperative plasma inflammatory cytokine concentrations are associated with increased CSA-AKI occurrence and mortality [5]. Reperfusion of hypoxic tissue after CPB produces ROS, which in turn promote inflammation by upregulating proinflammatory transcription factors [69], further exacerbating cellular dysfunction and renal injury [70].

Tissue hypoxia resulting from renal hypoperfusion due to decreased CO and GFR is also responsible for the inflammatory response and subsequent oxidative stress. Ischaemia causes tissue inflammation by causing endothelial cell damage [71]. The recruited leukocytes adhere to endothelial cells and cause endothelial damage, which in turn initiates a cascade of inflammatory responses [72]. This inflammatory cascade eventually leads to dysfunction of the renal endothelium nitric oxide system, which plays an important role in renal oxygen delivery [73]. Neutrophils, macrophages, and lymphocytes activated during inflammation migrate to the renal parenchyma and exacerbate renal injury, thereby promoting AKI and possibly causing fibrosis [74].

The CPB procedure exposes the blood components to shear stress and oxidative stress, causing the lysis of red blood cells and the release of free haemoglobin and iron [75]. The accumulated free iron is involved in pro-oxidative reactions that produce oxygen free radicals, leading to tissue damage. IRI may also exacerbate oxidative inflammatory stress during CPB, leading to an increase in circulating free labile iron [76]. Labile catalytic iron can damage renal epithelial cells, impair cell proliferation, and induce lipid peroxidation and protein oxidation [77].

### Nephrotoxins

Perioperative use of nephrotoxic drugs is common in patients undergoing cardiac surgery, including medications for comorbidities such as antibiotics, blood pressure medications, diuretics, nonsteroidal anti-inflammatory drugs (NSAIDs), and radiocontrast agents used in medical diagnostic procedures.

NSAIDs inhibit cyclooxygenase, which may lead to reversible renal ischaemia and decreased GFR, worsening renal function in susceptible individuals [78]. By blocking prostaglandin synthesis, the irrational use of NSAIDs has been shown to cause persistent severe outer medullary hypoxia [79].

Angiotensin-converting enzyme inhibitors (ACEis) and angiotensin receptor blockers (ARBs) can cause volume depletion and vasodilation of renal efferent arterioles, respectively [49], thereby promoting AKI [80]. Patients taking ARBs and diuretics in combination have an increased risk of hypovolemia, which exacerbates renal failure [81].

Aminoglycosides exhibit several favourable pharmacokinetic and pharmacodynamic properties, but dose-dependent nephrotoxicity may cause direct injury to the kidney, resulting in drug-induced AKI. The risk of AKI attributable to aminoglycosides can be sufficiently high [82], and perioperative use of gentamicin is associated with an increased risk of postoperative dialysis [83]. The KDIGO AKI Guideline Working Group recommends cautious use of aminoglycosides during the perioperative period [82].

Randomized controlled trials have shown that prophylactic use of cyclic diuretics, such as furosemide, during the perioperative period in patients planning cardiac surgery is ineffective and even harmful [84]. The KDIGO AKI Guideline Working Group recommends not using furosemide as a prophylactic for the prevention of AKI and avoiding diuretics in the treatment of AKI [82].

Contrast agents have been suggested to induce AKI in patients undergoing coronary angiography or percutaneous coronary intervention [85]. Ranucci et al. showed that restricting angiography on the day of surgery could reduce the incidence of AKI after cardiac surgery [86]. Performing cardiac surgery on the day of the catheterization procedure and administering a high-dose contrast agent are independently associated with an increased risk of postoperative AKI [87]. However, another study suggested that contrast therapy within 48 h before the start of CPB in patients with cyanotic congenital heart disease did not increase the risk of AKI [88]. Even though the results of clinical studies regarding the association between angiography and CSA-AKI are inconsistent, it is recommended to avoid angiography before cardiac surgery or to extend the interval between surgery and angiography. Contrast agent-induced AKI (CI-AKI) can cause direct renal tubular injury, but the underlying mechanism remain unclear, including direct cytotoxic effects and autocrine, paracrine and endocrine factors [89]. Lau et al. demonstrated that CI-AKI involves a Nod-like receptor pyrin containing 3 (NIrp-3)-dependent inflammatory response between resident and infiltrating renal phagocytes, requiring tubular reabsorption of contrast media via the brush border enzyme dipeptidase 1 [90]. Iodinated contrast agent is the main media of contrast-induced nephropathy, and its osmotic pressure is one of the influencing factors. Studies have confirmed that the risk of CI-AKI is lower in high-osmolar contrast than in low-osmolar contrast, but there is no significant difference between iso-osmolar contrast and low-osmolar contrast [91, 92].

### Metabolic and neurohormonal activation

Cardiac surgery stimulates the SNS and the hypothalamic‒pituitary‒adrenal axis, which in turn causes the release of neurohormonal agents, including epinephrine and norepinephrine, and may increase the production of vasopressin and endothelin-1 [93]. Studies have confirmed that plasma concentrations of epinephrine and norepinephrine reach peak levels during CPB cardiac surgery [94]. High plasma concentrations of these endogenous hormones cause unstable haemodynamic conditions and systemic vasoconstriction [93], which can lead to decreased renal perfusion and ultimately renal injury.

Free iron release is a common consequence of CPB. During CPB, when red blood cells come into contact with artificial surfaces or air, a degree of haemolysis is inevitable, and the accompanying prolonged cold temperatures provide the perfect environment for haemolysis and the release of free iron, leading to vasoconstriction through scavenging of nitric oxide by free haemoglobin [95]. Free iron-mediated toxicity may also be an important mechanism for AKI in patients undergoing CPB cardiac surgery. Several novel renal biomarkers have been implicated in iron metabolism, including NGAL, L-FABP, α-1 microglobulin, and hepcidin isoforms [96]. Free iron-related and ROS-mediated renal injury seems to be the unified pathophysiological association between these biomarkers. Iron regulation plays a role in the development of AKI after cardiac surgery and is associated with oxidative stress and IRI. Various prevention and treatment strategies related to iron regulation are being investigated for AKI [97]. Hepcidin is an endogenous acute-phase liver hormone that prevents iron export from cells by inducing the degradation of ferroportin, the only known iron export protein. Hepcidin-induced restoration of iron homeostasis was accompanied by significant reductions in ischaemia‒reperfusion-induced tubular injury, apoptosis, renal oxidative stress, and inflammatory cell infiltration [98]. Several small studies have investigated iron chelation as a novel therapeutic strategy for the prevention of AKI and have shown encouraging initial results [99].

### Genetic polymorphisms

A number of studies have suggested the role of genetic polymorphisms in the development of CSA-AKI. Polymorphisms of angiotensinogen, apolipoprotein E (APOE), angiotensin-converting enzyme (ACE), endothelial nitric oxide synthase (eNOS), erythropoietin, interleukin-6 (IL-6), interleukin-10 (IL-10), catechol-O-methyltransferase (COMT), GRM7 | LMCD1—AS1 loci and BBS9, transducer and activator of transcription 3 (STAT3), and macrophage migration inhibitory factor (MIF) genes have been proven to be associated with a higher risk of AKI after cardiac surgery [100]. However, some studies have denied a correlation between some of these gene polymorphisms and CSA-AKI [101, 102]. In the pathogenesis of AKI, various genes act together to generate favourable or harmful proinflammatory and anti-inflammatory cytokine environments, thereby determining the intensity of tissue damage. Genetic variations may affect how the kidney responds to injury, determining the outcome of the patient. Thus, knowledge of genetic polymorphisms can facilitate patient management by modifying risk stratification tools and detecting genetic susceptibility biomarkers [100].

## Strategies for CSA-AKI prevention

### Preoperative strategies

#### Pharmacological interventions

There is no specific pharmacological intervention to prevent CSA-AKI. Many perioperative pharmacologic prophylaxis strategies have been introduced, evaluated, and then abandoned [103, 104], such as perioperative use of corticosteroids, albumin, erythropoietin, statins, N–acetylcysteine, and sodium bicarbonate and intraoperative or postoperative use of dopamine, anaesthetics (volatile or intravenous), mannitol, furosemide, and fenoldopam [12, 103]. It is worth noting that most studies were small, used different inclusion criteria, administered different doses of drugs at different times, investigated different outcomes, and used different criteria to define AKI [105]. Considering that the relevant research evidence is weak, some studies are contradictory, and some drugs have even been proven to be potentially harmful in certain circumstances, none of these methods are routinely used. Efforts are still being made to identify potential drug prevention strategies for CSA-AKI, and the findings suggest several potentially promising drugs.

Dexmedetomidine is an α_2_-adrenergic receptor agonist with sedative, analgesic, and anti-sympathetic effects. Data from animal models suggest that pretreatment with dexmedetomidine before IRI has a nephroprotective effect by reducing the levels of inflammatory cytokines and damage associated molecular patterns (DAMPs), thereby reducing cell death and toll-like receptor 4 expression in renal tubular cells after renal ischaemia [106]. These results are also reflected in the data from clinical studies: several recent meta-analyses [104, 107] show the benefit of perioperative dexmedetomidine in reducing the incidence of CS-AKI, especially in elderly patients who received dexmedetomidine immediately after induction of anaesthesia and maintained intraoperative infusion. However, the subsequent DECADE trial suggested that perioperative use of dexmedetomidine tended to worsen delirium and AKI after cardiac surgery, although not by a significant amount [108]. The authors suggested that dexmedetomidine should be used cautiously in patients undergoing cardiac surgery, given the concerning incidence of serious hypotension events. Another meta-analysis, including the DECADE study, found that perioperative dexmedetomidine did not reduce the incidence of CSA-AKI [12].

Atrial natriuretic peptide (ANP) has been reported to inhibit the RAAS and SNS, both of which contribute to the pathophysiology of CSA-AKI. Earlier meta-analyses found that low-dose ANP reduced the need for RRT in patients undergoing cardiovascular surgery, whereas high-dose ANP was associated with more adverse events (hypotension, arrhythmia) [109]. A recent meta-analysis involving 228 trials (56047 patients) evaluated the effect of pharmacological interventions on postoperative renal protection. The study found that the use of ANP (13 trials in cardiac surgery and 1 trial in vascular surgery; 2207 patients) reduced 30-day mortality and AKI, with subgroup analyses demonstrating a significant treatment effect of ANP on AKI, mortality, and RRT in cardiac surgery cohorts [104].

Levosimendan is a calcium-sensitive inotropic vasodilator that is commonly used to treat low cardiac output after cardiac surgery and perioperative cardiovascular dysfunction. It is believed to have antioxidant, anti-inflammatory, and anti-apoptotic properties and has been found to significantly attenuate renal tubule IRI in animal models [110]. Two multicentre RCTs investigating the effect of levosimendan on cardiac surgery found no difference in the incidence of AKI or RRT [111, 112]. A recent meta-analysis assessed the effect of levosimendan on cardiac surgery outcomes in 13 trials with 941 participants, and levosimendan was found to reduce the need for postoperative RRT after excluding studies with a high or uncertain risk of allocation concealment [104].

#### Optimization of preoperative medication

Nephrotoxins should potentially be avoided during the perioperative period of cardiac surgery. The European Renal Best Practice (ERBP) Working Group does not oppose the use of aminoglycosides as a single dose, such as antibiotic prophylaxis for cardiac surgery. If more than one dose is needed, it is recommended that this group of antibiotics be used for as short a time as possible [113]. The opinion on the preoperative withdrawal time of ACEis/ARB drugs is still controversial. Although evidence is limited, some studies have shown that preoperative retention of ACEis/ARBs is associated with a reduced incidence of AKI [114]. For this reason, the 20th International Consensus Conference of the ADQI recommended withholding ACEis and ARBs during the perioperative period [115]. However, the 2021 joint consensus report of the ADQI and Perioperative Quality Initiative (POQI) recommends that ACEis and ARBs be discontinued at least 24 h before surgery to minimize the risk of perioperative hypotension and/or postoperative AKI [9]. Although this recommendation is specific to noncardiac surgery, it still has implications for CSA-AKI.

#### Prehabilitation

Both guidelines from American ERAS society [116] and French society of anaesthesia and intensive care medicine (SFAR) [117] recommend that prehabilitation be started at least 4 weeks before cardiac surgery to reduce the occurrence of postoperative complications and hospital stay. The cardiac prehabilitation program should include education, nutritional optimization, exercise training, social support, and anxiety reduction, which enables the patient to withstand the stress of surgery by enhancing functional capacity. The current evidence mainly focuses on the role of preoperative cardiopulmonary and muscle pre rehabilitation in reducing hospital stay and pulmonary complications after cardiac surgery [118]. Although there is evidence to support that prehabilitation exercise therapy before elective abdominal aortic aneurysm repair can reduce postoperative serum creatinine and the requirement for haemodialysis/haemofiltration [119], the role of prehabilitation in CSA-AKI remains uncertain.

### Intraoperative strategies

#### Optimal target of mean arterial pressure

Maintenance of renal perfusion pressure and renal blood flow is still the main focus of perioperative management in cardiac surgery. Decreased mean arterial pressure (MAP) and increased fluid balance were independently associated with increased mortality and the need for RRT after cardiac surgery [120]. In response to changes in MAP, renal autoregulation attempts to maintain renal blood flow and GFR by activating the RAAS and vasopressin secretion. The renal response to a state of low blood flow includes a decrease in renal blood flow, GFR, and urine output, as well as an increase in proximal tubule reabsorption [121]. The range of renal autoregulation differs between individuals. In today's practice, the provision of anaesthesia is best titrated according to consciousness or brain function monitoring combined with haemodynamic monitoring to ensure adequate anaesthesia and avoid hypotension.

The optimal target MAP in cardiac surgery has been under discussion. A recent retrospective study showed that MAP < 65 mmHg for more than 10 min after CPB was associated with an increased risk of new postoperative RRT [122]. However, subsequent meta-analyses did not find an association between hypotension before or during CPB and RRT, and the authors concluded that the association between intraoperative hypotension and AKI was weaker than that between pre-existing and surgical factors (e.g., obesity, anaemia, renal insufficiency, heart failure, complex or emergency surgery) [12]. The normal range of renal autoregulation is 75 to 160 mmHg, and MAP is usually below the lower limit of this range during CPB. However, in two RCTs, increasing MAP during CPB was not shown to be associated with a reduced incidence of AKI or chronic kidney injury [123]. In a trial comparing a high MAP target (70–80 mmHg) with a low MAP target (40–50 mmHg) during CPB, a significantly larger number of patients doubled postoperative SCr levels in the high target group [124]. It is suggested that the use of vasopressors in the pursuit of blood pressure increase during cardiac surgery may increase postoperative creatinine levels and that personalized blood pressure management may be most effective [12]. Interventions applied to patients with CSA-AKI, as well as assessment of treatment response, remain more of an art than a science, given the extraordinary complexity and moment-to-moment or patient-to-patient differences in CSA-AKI [37]. Currently, the recommendation from EACTS/EACTA/EBCP is to maintain a MAP target between 50 and 80 mmHg during CPB [125].

#### Inotropic and vasopressor support

Inotropic agents may be used to improverenal perfusion in cases of low cardiac output, and vasopressors may be used to increase renal perfusion pressure in cases of hypotension. Catecholamines (norepinephrine and epinephrine), vasopressin, dopamine, and angiotensin II all increase systemic vascular resistance and can be used to raise blood pressure. Vasoplegia is a common complication of CPB, and vasopressin was previously considered to be a reasonable first-line drug for the treatment of vascular paralysis after cardiac surgery [113]. A recent study by Hajjar et al. also found that vasopressin administration reduced the incidence of acute renal failure by 74% when compared with norepinephrine in patients with vasoplegic shock, defined as MAP < 65 mmHg, ineffective fluid resuscitation, and cardiac index > 2.2 L/min^2^/m^2^ [126]. However, the ADQI group in 2018, despite consideration of Hajjar’s article, recommended norepinephrine as the first choice for the treatment of vasoplegia during cardiac and vascular surgery and stated that more research was needed before supporting vasopressin as an equal choice to norepinephrine [113].

#### Fluid, diuretics and goal-directed therapy

Whilst maintaining renal perfusion by maintaining MAP and cardiac output, clinicians also seek to avoid hypotensive states by judicious use of intravenous fluids and antidiuretics. However, fluid management during and after cardiac surgery remains complex and controversial. Both excessive accumulation of extracellular fluid and intravascular hypovolemia can contribute to AKI [37]. Two single-centre cohort studies showed that higher positive fluid balance is associated with increased AKI or RRT requirements after cardiac surgery [120, 127]. Recently, a multicentre prospective study of intraoperative fluid balance and AKI after CABG found that patients receiving liberal fluid balance had a similar incidence of CSA-AKI but higher in-hospital mortality, cardiovascular complications, and length of hospital stay than patients receiving restrictive fluid balance [128].

Alternatively, the type of fluid is also associated with the risk of postoperative AKI. Trials have shown that fluid resuscitation with hydroxyethyl starch (HES) is associated with an increased risk of AKI. Therefore, the use of HES is not recommended in patients at high risk for CSA-AKI [129]. In terms of the choice of crystalloid, perioperative use of chloride liberal intravenous fluid was found to be associated with hyperchloremic metabolic acidosis and increased postoperative AKI in patients who underwent off-pump coronary artery bypass grafting surgery [130]. When compared with Ringer acetate, 4% albumin solution for priming CPB and perioperative venous volume replacement solution cannot reduce the incidence of CSA-AKI [131].

The perioperative use of diuretics is common in cardiac surgery. Some trials have demonstrated adverse effects of diuretic use. The results of a recent meta-analysis suggested that loop diuretics (furosemide) may reduce postoperative creatinine clearance, whereas aldosterone agonists (spironolactone) increase the incidence of CSA-AKI [104]. Diuretics are not recommended for the prevention of AKI, but loop diuretics may be used for the management of volume overload [8].

Goal-directed therapy is a strategy to increase cardiac output by using fluid and/or contractile drugs to improve oxygen supply to organs and peripheral tissues, which has been shown to have a nephroprotective effect and can reduce the incidence of AKI after cardiac surgery [132]. Goal-directed therapy during cardiac surgery has been suggested as a class I recommendation (evidence and/or general agreement that a given treatment or procedure is beneficial, useful and effective) with evidence of level A (data derived from multiple RCTs or meta-analyses) in guidelines published by the EACTS/EACTA/EBCP in 2019 [125].

#### Goal-directed oxygen delivery in CPB

In addition to renal perfusion pressure, pump flow and flow-related oxygen delivery are also important factors in the prevention of CSA-AKI. Oxygen delivery during CPB has been shown to be directly related to postoperative AKI. The concept of goal-directed oxygen delivery (GDP) refers to maintaining oxygen delivery above the critical value during CPB, and the recommended critical value during moderate hypothermia CPB is 260–272 mL/min/m^2^ [59]. Several observational studies have proposed inadequate oxygen supply during CPB as a potential modifiable risk factor for AKI development [59, 133]. Based on these findings, the EACTS/EACTA/EBCP guidelines proposed that the pump flow rate should be adjusted according to the arterial oxygen content to maintain a minimal threshold of DO_2_ under moderate hypothermia as a class IIa recommendation (weight of evidence/opinion is in favour of usefulness/efficacy) [125]. Subsequently, Ranucci et al. conducted a multicentre randomized trial to compare the effects of a GDP strategy during CPB (maintaining DO_2_ ≥ 280 mL/min/m^2^) with a control perfusion strategy (calculated in reference to body surface area and temperature) on the incidence of AKI after cardiac surgery. The results showed that the GDP strategy was effective in reducing AKIN stage 1 AKI [134]. Recently, a similar study by Mukaida et al. demonstrated that the GDP strategy (maintaining DO_2_ > 300 mL/min/m^2^) during CPB reduced the incidence of AKI when compared with fixed flow perfusion. Notably, both trials reported that GDP during CPB reduced postoperative AKI by approximately 50%. A recent meta-analysis comprehensively evaluated these two RCTs and ultimately recommended the use of GDP to prevent CSA-AKI during CPB, despite concerns about limitations such as sample size, quality, and variation in intervention protocols [12].

#### Transfusion of packed red blood cells

Patients undergoing cardiac surgery are at higher risk of haemodynamic dysfunction and bleeding during surgery, which is also a predisposing factor for renal hypoxia. Preoperative anaemia is associated with AKI and mortality after cardiac surgery, but intraoperative infusion of packed red blood cells has also been identified as a risk factor for CSA-AKI [135], making it critical to determine the optimal threshold for intraoperative erythrocyte transfusion. A restrictive transfusion strategy (maintaining haematocrit (Hct) ≥ 24%) was found to have noninferior 30-day mortality and renal complication outcomes when compared with a liberal transfusion strategy (maintaining Hct ≥ 30%) [136]. Subsequently, several large RCTs have compared the restrictive transfusion threshold with the liberal transfusion threshold, but failed to report any differences with regard to AKI in both groups [137, 138]. The above results are also supported by a recent meta-analysis [139]. Available evidence suggests that restrictive transfusion thresholds have no effect on the incidence of CSA-AKI. Current guidelines recommend infusion of packed red blood cells if Hb < 6.0 g/dL [140], and an acceptable HCT value is between 21 and 24% when DO_2_ > 273 mL/min/m^2^ [141]. In addition, the storage time of red blood cells did not appear to be significant for the incidence of CSA-AKI [142].

#### Remote ischaemic preconditioning

Remote ischaemic preconditioning (RIPC) is a technique that artificially induces repeated transient ischaemia‒reperfusion of the distal limb, aiming to protect against subsequent ischaemic damage to sensitive organs and tissues. The specific operation is to keep a blood pressure cuff on the upper arm or thigh inflated for a few minutes, release the cuff, and then repeat the above operation.

Although the mechanism remains unclear, it has been suggested that the ischaemia‒reperfusion process stimulates the release and activation of anti-inflammatory cytokines, hypoxia-inducible factors, and neural autonomic and humoral signalling pathways, thereby preventing and attenuating distal organ dysfunction. A potential nephroprotective effect is mediated by the release of DAMPs, which activate toll-like receptors in the proximal tubular epithelium [143], thereby inducing natural defences, including bioenergetic downregulation and temporary cell cycle arrest, and protecting the kidney during subsequent stress [144].

The role of RIPC in preventing CSA-AKI remains controversial. Although a small single-centre RCT demonstrated that RIPC significantly reduced the incidence of AKI after cardiac surgery and the need for RRT [145], two large multicentre RCTs found no difference in the incidence of AKI after cardiac surgery [146, 147]. Several studies have suggested that propofol may reverse the effects of RIPC [148, 149]. Interestingly, the results of two meta-analyses also suggested that RIPC could reduce the incidence of postoperative AKI in patients undergoing cardiac surgery in a subgroup of volatile anaesthetics [150, 151]. As the mechanism of action and its potential interference are not fully understood, further research is warranted to identify specific patients and conditions that could benefit from this technique.

### Postoperative strategies

#### KDIGO bundle of care

The pathophysiology of CSA-AKI is complex, making it difficult for a single intervention to achieve substantial renal protection. A series of consensus/guideline-based interventions in patients at risk for AKI may be more likely to provide significant improvement for postoperative renal outcomes. The 2012 KDIGO guidelines recommend a variety of supportive measures, including discontinuation of nephrotoxins if possible, optimization of fluid status and haemodynamics, consideration of functional haemodynamic monitoring, monitoring SCr and urine output, avoidance of hyperglycaemia and consideration of alternatives to radiocontrast procedures [82].

Two RCTs investigated whether the KDIGO bundle of care impacted the occurrence of CSA-AKI in high-risk patients. A single-centre study by Meersch et al., involving 276 patients, showed that this KDIGO bundle strategy significantly reduced the incidence and severity of AKI in patients at high risk for CSA-AKI [152]. A recent multicentre study by Zarbock et al., involving 278 patients, showed that the incidence of stage 2 and 3 AKI was significantly lower in the KDIGO bundle group [25]. In their study, however, compliance with the KDIGO bundle was notably low: 65.4% of the patients in the intervention group, compared with 4.2% of those in the control group, received a complete bundle. This was also confirmed by a later observational study involving 12 hospitals in different European countries, in which only 5.3% of patients after cardiac surgery complied with all the items recommended by KDIGO in routine clinical practice, with an average of 3.4 out of 6 items received in each high-risk patient [153]. A recent meta-analysis showed that the use of the KDIGO bundle of care did not reduce the overall incidence of AKI in high-risk patients but significantly reduced the incidence of stage 2 or 3 AKI, and use of the KDIGO bundle of care was recommended to prevent CSA-AKI in high-risk patients, although larger trials are still needed [12]. Similarly, it is recommended in the guidelines for perioperative care in cardiac surgery by the ERAS Society that the implementation of the KDIGO bundle of care in high-risk patients can reduce the incidence of stage 2 or 3 AKI [116].

## Conclusion and perspective

AKI associated with cardiac surgery is a complex multifaceted syndrome with high morbidity and mortality. Early identification of high-risk patients, early diagnosis of AKI, emphasis on prevention, avoidance of nephrotoxins, attention to optimization of systemic haemodynamics, maintenance of oxygen supply, and reasonable management of fluid are still the main aspects of the current clinical management of CSA-AKI. In terms of CSA-AKI diagnosis, despite of constantly arising of new biomarkers, predictive models and diagnostic methods such as point-of-care ultrasound etc. However, current diagnostic modalities have not made significant improvement in early diagnosis. Therefore, further exploration for prospective methods of early identification is essential and urgent needed. In addition, the performance of present medications or interventions in preventing CSA-AKI are less satisfactory. Therefore, further clinical trials to explore and verify the potential pharmacologic or non-pharmacologic preventive strategies remain a priority in this area.

## Data Availability

Not applicable.

## Abbreviations

ACE: Angiotensin-converting enzyme
ACEis: Angiotensin-converting enzyme inhibitors
ADQI: Acute dialysis quality initiative
AKI: Acute kidney injury
AKIN: Acute kidney injury network
ANP: Atrial natriuretic peptide
APOE: Apolipoprotein E
ARBs: Angiotensin receptor blockers
ARF: Acute renal failure
CABG: Coronary artery bypass grafting
CI-AKI: Contrast induced-AKI
CKD: Chronic kidney disease
COMT: Catechol-O-methyltransferase
CPB: Cardiopulmonary bypass
CRS: Cardiorenal syndrome
CSA-AKI: Cardiac surgery-associated acute kidney injury
DAMPs: Damage-associated molecular patterns
DECADE trial: Dexmedetomidine for reduction of atrial fibrillation and delirium after cardiac surgery
DKK3: Dickkopf-3
DO_2_: Oxygen delivery
EACTS/EACTA/EBCP: European association for cardiothoracic surgery/European association of cardiothoracic anaesthesiology/European board of cardiovascular perfusion
eNOS: Endothelial nitric oxide synthase
ERAS: Enhanced recovery after surgery
ERBP: European renal best practice
GDP: Goal-directed oxygen delivery
GFR: Glomerular filtration rate
HCT: Haematocrit
HES: Hydroxyethyl starch
ICU: Intensive care unit
IGFBP7: Insulin-like growth factor-binding protein 7
IL-10: Interleukin-10
IL-18: Interleukin-18
IL-6: Interleukin-6
IRI: Ischaemia–reperfusion injury
KDIGO: Kidney disease: improving global outcomes
KIM-1: Kidney injury molecule 1
L-FABP: Liver fatty acid‐binding protein
MAP: Mean arterial pressure
MIF: Macrophage migration inhibitory factor
NGAL: Neutrophil gelatinase-associated lipocalin
NIrp-3: Nod-like receptor pyrin containing 3
NSAIDs: Nonsteroidal anti-inflammatory drugs
RAAS: Renin–angiotensin–aldosterone system
RCT: Randomized controlled trial
RIPC: Remote ischaemic preconditioning
ROS: Reactive oxygen species
RRT: Renal replacement therapy
SCr: Serum creatinine
SNS: Sympathetic nervous system
STAT3: Transducer and activator of transcription 3
TIMP-2: Tissue inhibitor of metalloproteinases-2
UO: Urine output
USD: United States dollar
